# Isolation of* Helicobacter pylori* from Gastric Biopsy of Dyspeptic Patients in Ghana and* In Vitro* Preliminary Assessment of the Effect of* Dissotis rotundifolia* Extract on Its Growth

**DOI:** 10.1155/2018/8071081

**Published:** 2018-10-31

**Authors:** Michael Buenor Adinortey, Charles Ansah, Cynthia Ayefoumi Adinortey, Ansumana Sandy Bockarie, Martin Tangnaa Morna, Damian H. Amewowor

**Affiliations:** ^1^Department of Biochemistry, School of Biological Sciences, College of Agriculture and Natural Sciences, University of Cape Coast, Cape Coast, Ghana; ^2^Department of Pharmacology, Faculty of Pharmacy and Pharmaceutical Sciences, Kwame Nkrumah University of Science and Technology, Kumasi, Ghana; ^3^Department of Molecular Biology and Biotechnology, School of Biological Sciences, College of Agriculture and Natural Sciences, University of Cape Coast, Cape Coast, Ghana; ^4^Department of Internal Medicine, School of Medical Sciences, College of Health and Allied Sciences, University of Cape Coast, Cape Coast, Ghana

## Abstract

*Helicobacter pylori* (*H. pylori*) is a gram-negative bacterium that colonizes the human stomach. Infection with this microaerophilic bacterium causes gastric and duodenal ulcer. This study sought to isolate* H. pylori*, from gastric biopsy samples of dyspeptic patients in Ghana using a 2,3,5-triphenyltetrazolium chloride (TTC) dye incorporated medium method. This TTC dye method was further used in an antimicrobial susceptibility assay involving* Dissotis rotundifolia* extract (DRE).* H. pylori* were successfully isolated from gastric biopsy of dyspeptic patients. Pure cultures of* H. pylori* in 2,3,5-triphenyltetrazolium chloride (TTC) dye incorporated medium were seen as sparkling colonies. Isolates, identified as* H. pylori*, were gram-negative and urease, catalase, and oxidase positive and showed characteristic morphology as spiral-shaped bacteria under the microscope. The organisms were found to be susceptible to cephalothin and resistant to nalidixic acid. Above all, the observation that* H. pylori* grew only at 37°C and not 25°C or 42°C affirms that the bacterium is neither* Helicobacter cinaedi* nor* Helicobacter fenneliae.* The anti-*H. pylori* study depicts a statistically lower zone of inhibition for DRE compared to standard drugs [amoxicillin and clarithromycin] (p<0.05), whereas metronidazole showed no zone of inhibition. This study reports the first successful isolation and culturing of* H. pylori* in Ghana using TTC dye. It also shows that DRE possess an* in vitro* anti-*H. pylori* activity and that DRE has some therapeutic potential against* H. pylori* infection.

## 1. Introduction

The discovery of* Helicobacter pylori *(*H. pylori*) in 1982 [1] was the beginning of a transformation concerning the management of gastroduodenal diseases. The public health relevance of the discovery and its role in gastrointestinal diseases was recognized in 2005 by the credit of the Nobel Prize in Physiology or Medicine to B. Marshall and R. Warren [2].* H. pylori* is a gram-negative bacterium that colonizes the human stomach. Infection with this gram-negative organism is linked with gastritis and peptic ulcer diseases which when improperly managed may eventually result in the development of gastric adenocarcinoma and mucosa-associated lymphoid tissue (MALT) lymphoma [3]. The invasion of the stomach by this fastidious bacterium results in an imbalance between aggressive and defense factors. Among peptic ulcer patients,* H. pylori* is reported to be the main etiological agent with an overall prevalence rate of about 25-30 % in developed countries, and over 80 % in developing countries in peptic ulcer patients [4].* H. pylori* is reported to be a major cause of at least 90 % of duodenal ulcers and 70 % of gastric ulcers [5]. Eradication of* H. pylori *has been reported to result in peptic ulcer healing, prevention of recurrence, and reduction in the prevalence of gastric cancer in high-risk populations [6].

Though several studies have been conducted on* H. pylori *in Ghana [7–12], no report is available on the use of the culture method. The unsuccessful attempts to use the culture technique have made it impossible to deploy antimicrobial susceptibility assay methods and also characterize* H. pylori *strains in Ghana. This for a long time has slowed down research involving the use of* H. pylori* and associated infections in Ghana. This challenge has made it virtually impossible to obtain information regarding drug resistance profile of* H. pylori* in Ghana, which has slackened research progress in healthcare for peptic ulcer patients. This study sought to isolate* H. pylori *from gastric biopsy samples of dyspeptic patients by using a modified culturing technique involving 2,3,5-triphenyltetrazolium chloride (TTC) dye. The adaptability of this culturing method was subsequently tested in a susceptibility assay to determine the anti-*H. pylori *effects of extracts of* Dissotis rotundifolia* on clinical isolates using the agar well diffusion assay method.

## 2. Materials and Methods

### 2.1. Chemicals, Drugs, and Other Consumables

The following chemicals, drugs, and test kits were used for the experiments: urease test kit (ClO test) was obtained from USA. Brain Heart Infusion (BHI) agar, Brain Heart Infusion (BHI) broth, Skirrow's supplement (SR69) Oxoid England, glycerol, cephalothin (30 *μ*g), and nalidixic acid (30 *μ*g) antibiotic disc were obtained from Mast Group Ltd., Merseyside, UK. GasPak EZ Campy Container System was obtained from Becton Dickinson, USA. Sodium hydroxide, magnesium chloride, sucrose, glucose, methanol (99.8%), and ethanol (99.8%) were obtained from British Drug House, (BDH) (London, UK). 2,3,5-Triphenyltetrazolium chloride (TTC) was obtained from Sigma Aldrich. Expired blood was obtained from the Blood Bank of the Cape Coast Regional hospital, Cape Coast, Ghana.

### 2.2. Collection of Plant Material and Extraction Method

*Dissotis rotundifolia* whole plant was collected from the environment around the Kakum National Park, Cape Coast, Ghana, and validated by a curator at the Herbarium of the University of Cape Coast. A voucher specimen (No. 107346) was prepared and deposited at the herbarium. The whole plant was thoroughly washed, shade-dried for three weeks, oven-dried at 40°C for 4 h, and then milled into powder. The method of extraction described by Rath et al. [13] involving the sequential use of dichloromethane and 70% methanol was employed in the preparation of the crude whole plant extract. The obtained crude DRE was stored at −20°C until it was ready for use.

### 2.3. Preparation of Media

*Christensen's Urea*. Peptone (0.08 g), 0.4 g NaCl, 0.16 g H_2_PO_4_, 0.08 g D-glucose, and 1.6 g of agar powder were transferred into a conical flask. Distilled water (72 mL) was added and the mixture was microwaved to ensure complete dissolution. It was then allowed to cool sufficiently to 50°C before phenol red (480 *μ*L) was added. The pH of the mixture was adjusted to 6.30 and the medium was autoclaved at 121°C for 15 minutes. Urea (1.6 g) was dissolved in 8 mL of sterile distilled water and added to the medium. The medium was poured aseptically into test tubes, allowed to solidify in a slanted position, and stored.

*Amended Brain Heart Infusion Blood Agar. *Dehydrated powdered Brain Heart Infusion agar (5.20 g) was transferred into a 250 mL Erlenmeyer flask. Distilled water (100 mL) was added. The mixture was heated briefly in a microwave until the agar was completely dissolved. The flask was corked with cotton wool plug and it was autoclaved at 121°C for 15 minutes and allowed to cool to approximately 50°C. Expired human blood (7 mL) was then added to the Brain Heart Infusion agar and 0.1 g of tetrazolium salt dissolved in 10 mL sterile distilled water was added. Skirrow's supplement (SR69) (Oxoid, England), vancomycin (5 mg), polymyxin B (2.5 mg), and trimethoprim (2.5 mg) were also added to the medium to suppress the growth of other bacteria and fungi. The mixture was swirled and was poured aseptically into sterile Petri dishes. The agar plates were then allowed to solidify and later kept in a refrigerator.

*Brain Heart Infusion Broth as Transport Medium. *Brain Heart Infusion (BHI) broth was used as the transport medium for the transportation of gastric biopsy samples obtained from the Endoscopy Laboratory. Broth was prepared by transferring 4.2624 g of the Brain Heart Infusion broth powder into a clean conical flask and 114.48 mL of sterile distilled water was added. Glycerol (28.8 mL) representing about 20 % of total solution was added to the solution and swirled until it was uniformly mixed. Cysteine solution (0.72 mL) of 0.2 g/L prepared by dissolving 0.5 g of cysteine powder in 2.5 mL of 1N HCl was also added to the mixture and the pH of the mixture was adjusted to 7.40. The BHI broth was then transferred into McCartney's bottles. Each of the bottles containing 18 mL of the broth was autoclaved at 121°C for 15 minutes and stored in a refrigerator.

*Brain Heart Infusion Broth for Antimicrobial Assay. *The broth was prepared by transferring 2.96 g of the Brain Heart Infusion (BHI) broth powder into a clean conical flask and adding 76 mL of distilled water. It was then autoclaved at 121°C for 15 minutes and allowed to cool. Sterile inactivated human serum (4 mL) representing 5% of medium was added and the mixture swirled until it was uniformly mixed. Skirrow's reagent was also added as a supplement. Then 5 mL of the mixture was transferred into clean test tubes that were corked with cotton wool and stored at -20°C until it was needed. Sterility quality control was performed by incubating an aliquot of the broth mixture at 37°C for 48 hrs.

### 2.4. Isolation of H. pylori

*Collection and Transportation of Gastric Biopsy*. Antral and corpus gastric mucosal biopsy specimens were taken from dyspepsia patients referred to Life Science Laboratory, Cape Coast, for endoscopy after informed consent was obtained from patients and approval of proposal by the Institutional Review Board of the University of Cape Coast. Biopsies confirmed to be positive for* H. pylori *using urease test kit were immediately placed in sterile McCartney bottles containing 0.2 g/L of cysteine and 20 % glycerol in brain heart infusion (BHI) broth and transported on ice to the laboratory within 60 minutes of collection for subsequent analysis.

*Inoculation of Gastric Biopsy. *The BHI broth containing the gastric biopsy was homogenized using the WiseTis homogenizer HG–15D, Germany, at 5000 rpm for 30 seconds to produce an evenly distributed mixture of biopsy tissue in the broth. The homogenized biopsy was then inoculated onto the brain heart infusion blood-amended agar. The inoculation was done, by dipping a flamed inoculation loop that has cooled for some time into the McCartney's bottle containing the homogenized biopsy tissue. The loop was used to streak the surface of an amended Brain Heart Infusion blood agar plate. The inoculation loop was flamed until they were red-hot after usage. The inoculated plates were then packed in an inverted manner into gaspak air-tight container and a gaspak kit that generates microaerophilic conditions (80 % N_2_, 10 % CO_2_, and 5 % O_2_) for the growth of* H. pylori *was included in the container. The replicated inoculated plates together with the gas generating kit were incubated at 25°C, 37°C, and 42°C for 3-7 days.

*Subculturing of Bacterial Isolates. *After 3-7 days of incubation, sparkling colonies were only observed on the amended BHI blood agar cultured at 37°C. A flamed inoculation loop was used to pick a single sparkling colony and streaked on the surface of a freshly amended BHI blood agar plate and incubated under microaerophilic conditions at 37°C for another 3-7 days to obtain pure cultures of* H. pylori*.

### 2.5. Microscopy and Biochemical Tests for Identification of H. pylori

*H. pylori *isolates were identified based on colony morphology and physical characteristics in the presence of TTC dye. Biochemical tests, namely, gram staining reaction, oxidase, urease, and catalase tests, and antibiotics (cephalothin and nalidixic acid) susceptibility test were employed [14, 15].

*Susceptibility Test for H. pylori Using Nalidixic Acid and Cephalothin Antibiotics. *A bacterial suspension was prepared by using a sterile inoculation loop to pick 3 discrete colonies of pure culture of* H. pylori *growing on BHI blood agar amended with Skirrow's supplement and TTC dye. These colonies were transferred into a test tube containing 5 mL of sterile normal saline and the bacterial suspension was briefly vortexed to ensure even distribution of bacteria.

About 0.1 mL of the* H. pylori *bacterial suspension was transferred unto the surface of an amended BHI blood agar plate and spread over the entire surface of the agar plate. With the aid of sterile forceps, cephalothin and nalidixic acid discs were placed on the surface of inoculated plate and incubated at 37°C for 3-6 days under microaerophilic conditions.

### 2.6. Assessment of DRE for Anti-H. pylori Activity

Bacterial suspensions of* H. pylori *were adjusted to the McFarland turbidity standards corresponding to 1.5x10^8^ CFU/mL. Agar wells were punched in sterile amended BHI blood agar plates using a sterile stainless 6 mm cork borer and allowed to dry for 3-5 minutes. The wells were then filled with 65 *μ*L each of different concentrations of the DRE (200, 400, 600, and 800 mg/mL), while distilled water was used as negative control and 0.05*μ*g/mL clarithromycin, 0.05 *μ*g/mL amoxicillin, and 500 *μ*g/mL metronidazole were used as positive controls. The inoculated plates were incubated at 37°C for 3-6 days under microaerophilic condition. After the incubation period, the diameters of the clear zones of inhibition around the agar wells were measured. The tests were done in triplicate. The mean zones of inhibition were calculated and presented as bar graphs.

### 2.7. Data Analysis

Statistical analysis was performed on data using the GraphPad prism version 6.0. ANOVA was used to determine differences in mean values and also if there was any statistically significant difference in the diameter of zones of inhibition of the DRE samples and antibiotics. Values were considered significant when* P*<0.05 followed by Bonferroni's pairwise post hoc test where there was a significant difference among means.

## 3. Results

**Figure 1:**
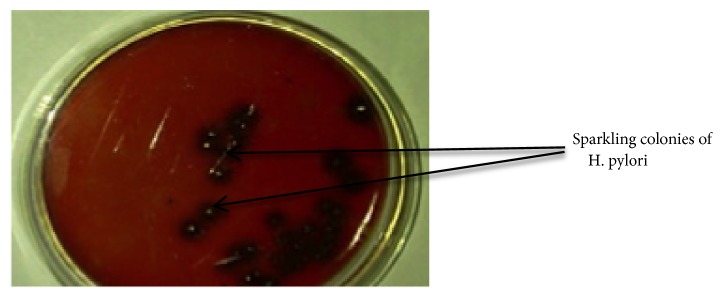
*H. pylori *sparkling colonies on BHI agar incorporated with TTC dye.

**Figure 2:**
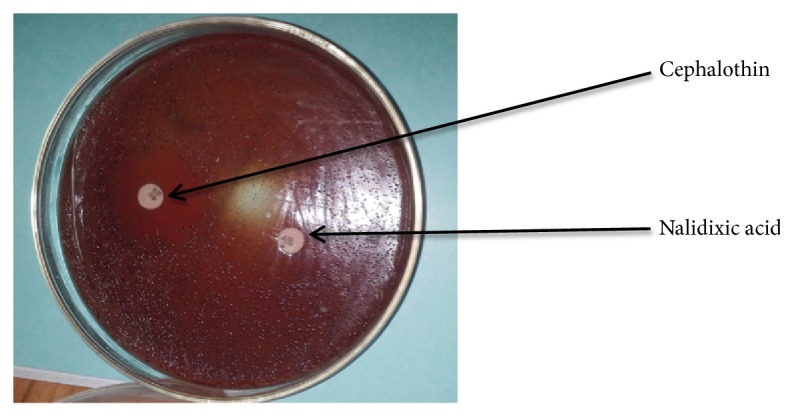
Susceptibility test for* H. pylori *using nalidixic acid and cephalothin antibiotic disc.

In this study,* H. pylori *were successfully isolated from the gastric biopsy of dyspepsia patients. Pure cultures of* H. pylori *in TTC dye incorporated medium were obtained as sparkling colonies (Figure 1). Isolates, identified as* H. pylori*, were gram-negative and urease, catalase, and oxidase positive and showed a characteristic morphology under the microscope. The organisms were also found to be susceptible to cephalothin and resistant to nalidixic acid (Figure 2).

**Figure 3:**
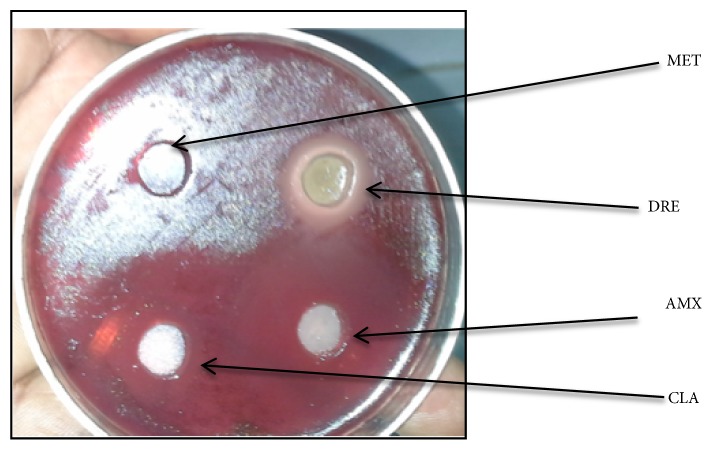
Inhibitory effects of* Dissotis rotundifolia* extract and standard antibiotics on growth of* H. pylori *on BHI agar.* Dissotis rotundifolia* extract (DRE), amoxicillin (AMX), clarithromycin (CLA), and metronidazole (MET).

**Figure 4:**
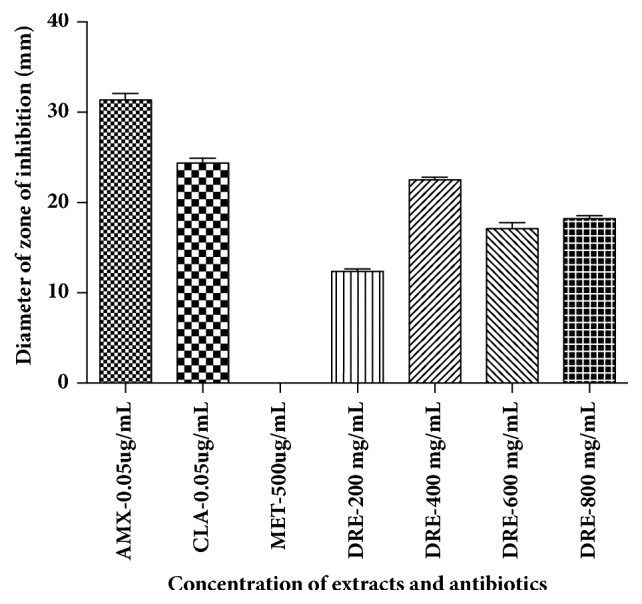
Anti-*H. pylori *activity of* Dissotis rotundifolia* extract and antibiotics. DRE,* Dissotis rotundifolia *extract; CLA, clarithromycin; AMX, amoxicillin; MET, metronidazole.

The whole plant of* Dissotis rotundifolia *extract (DRE) was screened for its potential in inhibiting growth of* H. pylori*. DRE and standard antibiotic drugs [amoxicillin (AMX), clarithromycin (CLA)] showed antimicrobial activity against 5 confirmed* H. pylori *isolates (Figure 3). The diameters of the zones of inhibition ranged from 13 to 30 mm (Figure 4). Autoclaved double distilled water used as negative control showed no inhibitory effect or activity. DRE showed significant lower zones of inhibition compared to standard drugs (clarithromycin, amoxicillin) (p<0.05). It was observed that metronidazole revealed no zone of inhibition. DRE has an inhibitory effect on growth of* H. pylori*. The inhibitory effect of DRE was observed from a concentration of 200 mg/mL to 800 mg/mL; meanwhile maximal effect was observed at 400 mg/mL (Figure 4).

## 4. Discussion

*H. pylori* is a major aetiological agent of peptic ulcer that is reported to have developed resistance against some antibiotics in many continents including Africa [5, 16, 17]. The several failed attempts in Ghana to culture the organism have made it difficult to use antimicrobial susceptibility assay and thus generate data on* H. pylori *resistance profile. To the best of our knowledge, this is the first report on a successful culturing and isolation of* H. pylori *from gastric biopsy in Ghana. It is also the foremost data on the activity of* Dissotis rotundifolia* extract against clinical isolates of* H. pylori*.

*H. pylori* bacterium can be distinguished from other species due to its multiple sheathed flagella, strong hydrolysis of urea, and unique fatty acid profile. This organism is one of the few that shows sparkling colonies when cultured on blood amended-Brain Heart Infusion agar incorporated with TTC dye. This characteristic was adopted in the isolation and culturing of the organism. The observation that* H. pylori *grew only at 37°C and not 25°C or 42°C affirms that the organism is neither* Helicobacter cinaedi* nor* Helicobacter fenneliae *as reported by Balows et al. [18].

Over the years, many phytomedicines have been used to treat infections due to* H. pylori *particularly in the developing world where this disease is endemic and modern health facilities and services are inadequate. The use of natural products is an attractive alternative treatment regime for* H. pylori *infected individuals. Garlic, honey, and many plant natural products have been found to be valuable in the treatment of* H. pylori *infections [19–24]. One such plant currently used is* Dissotis rotundifolia*. The whole plant of* Dissotis rotundifolia*, also known as pink lady, is popularly used for treatment of gastric disorders and other diseases. The plant according to Ansah et al. [25] is nontoxic to delicate organs such as the liver, kidney, and spleen of rats at a dose of 1000 mg/kgbwt. This plant has been reported to inhibit growth of* Staphylococcus aureus*,* Salmonella typhi*,* Escherichia coli*,* Pseudomonas aeruginosa*, and other organisms [26]. The ethnomedicinal report on the use of this plant in managing peptic ulcer and the evidence-based data on the antimicrobial activity of the extract on other gastrointestinal tract causing organisms prompted primary investigation into the potential effect of the extract on the growth of* Helicobacter pylori.*

As part of this research, a flavonoid-rich fraction of* Dissotis rotundifolia *was screened for its anti-*H. pylori *potential in an* in vitro* study. Although the results in this study depict statistically lower zones of inhibition for DRE compared to standard drugs [amoxicillin and clarithromycin] (p<0.05), it corroborates the reported ethnomedicinal use of the plant in managing gastric disturbances. The smaller zone of inhibition demonstrated by the flavonoid rich fraction of this plant* in vitro*, compared to standard antibiotic drugs, does not necessarily imply that the extract demonstrates weak antimicrobial effects* in vivo*. As with some orthodox drugs, the active ingredients in* Dissotis rotundifolia* may be pro-drugs; thus its potency could improve* in vivo *due to biotransformation [27].

C-glycosylflavones compounds, namely, vitexin and isovitexin, have been isolated from the methanolic extract of the whole plant of* Dissotis rotundifolia *[13]. Vitexin and isovitexin have also been isolated from the leaves of* Piper carpunya *and are reported to possess pharmacological activity against growth of* H. pylori *[28]. Implicitly the presence of these compounds in* Dissotis rotundifolia *whole plant extract may be contributing to the inhibitory effects of the plant in this study. The observation that* H. pylori *clinical isolates in this study showed resistance to metronidazole is not contrary to what has been reported in South Africa, Egypt, and many other African countries [5, 16, 17, 29]. This seems to corroborate an unreported emerging phenomenon of* H. pylori *resistance to metronidazole in Ghana and thus calls for an in-depth study of the pattern of drug resistance by* H. pylori* in Ghanaian dyspeptic patients.

In conclusion, this study reports on a first successful isolation of* H. pylori *from Ghanaian dyspeptic patients. It also depicts the fact that* Dissotis rotundifolia *extract possesses inhibitory potential against growth of clinical isolates of* Helicobacter pylori*. This finding suggests that* Dissotis rotundifolia extract* has some therapeutic potential against* H. pylori *infection, which could be explored in managing gastrointestinal problems.

## Data Availability

The data used to support the findings of this study are available from the corresponding author upon request.
